# Rabbit models of hip dysplasia: a narrative review

**DOI:** 10.1186/s42826-026-00291-9

**Published:** 2026-08-31

**Authors:** Jessica E. Goetz, Joshua Hockman, Michael C. Willey

**Affiliations:** 1 Department of Orthopedics & Rehabilitation, University of Iowa Healthcare Medical Center North Liberty, 701 Forevergreen Road, North Liberty, IA 52317 United States; 2 Department of Biomedical Engineering, University of Iowa, Iowa City, IA 52242 USA

**Keywords:** Hip, Hip dysplasia, Osteoarthritis, Rabbit, Animal model

## Abstract

The natural history of osteoarthritis development in individuals with hip dysplasia diagnosed after skeletal maturity is difficult to predict. While reorienting acetabular osteotomy improves pain and function for those with pre-arthritic hip dysplasia, the impact of dysplastic deformity correction on cartilage health and future cartilage degeneration remains unknown. The rabbit has been used as a preclinical model of hip dysplasia for investigation of joint degeneration and surgical treatment, but there is variability among protocols used to induce and characterize dysplastic deformity. The aim of this narrative review was to summarize techniques for inducing hip dysplasia, the associated morphologic and histopathologic changes, and the ability of surgical correction to mitigate joint degeneration in order to guide future research using the rabbit as a model of hip dysplasia. We identified 21 studies that induced dysplastic deformity in the rabbit hip. The most common method to induce dysplastic deformity was immobilization of the knee in extension in skeletally immature rabbits. Immobilization initiated at a younger age and for longer duration resulted in more severe dysplasia. In the few studies that assessed surgical treatment of the dysplastic hip, cartilage degeneration was reduced with correction of dysplastic deformity. We concluded that immobilizing skeletally immature rabbit knees in extension reliably causes dysplastic deformity of the hip, replicating a shallow and abnormally oriented acetabulum, which can progress to full dislocation more characteristic of hip dysplasia in infants and children, or be present without complete femoral head dislocation more characteristic of human hip dysplasia that presents in young adults. This model thereby offers the opportunity to assess the tissue level effects of hip dysplasia and the effects of surgical correction across the age spectrum.

**Clinical trials number **: Not applicable.

## Background

Hip osteoarthritis (OA) is a major cause of pain and disability, and responsible for most of the 2.3 million Americans currently living with a hip replacement [1]. Mounting evidence that pathologic joint mechanics due to hip dysplasia and/or femoroacetabular impingement are a cause of many hip OA cases previously classified as “primary,” particularly in individuals younger than age 50 years [2], has made it more common for surgical procedures such as hip arthroscopy and periacetabular osteotomy (PAO) to be performed to improve hip joint mechanics. Although the expectation is that correcting abnormal joint anatomy and mechanics will reduce future risk of developing OA, the impact of these surgical procedures on future OA development is unclear [3]. Key challenges of investigating the efficacy of surgical procedures to reduce hip OA progression in dysplastic hips include the inability to withhold surgical treatment for symptomatic patients, and the significant time between onset of symptoms and development of OA. Furthermore, while the natural history of developmental hip dysplasia diagnosed before skeletal maturity is well established [4], the natural history of osteoarthritis development is less clear when dysplasia is diagnosed after skeletal maturity.

**Fig. 1:**
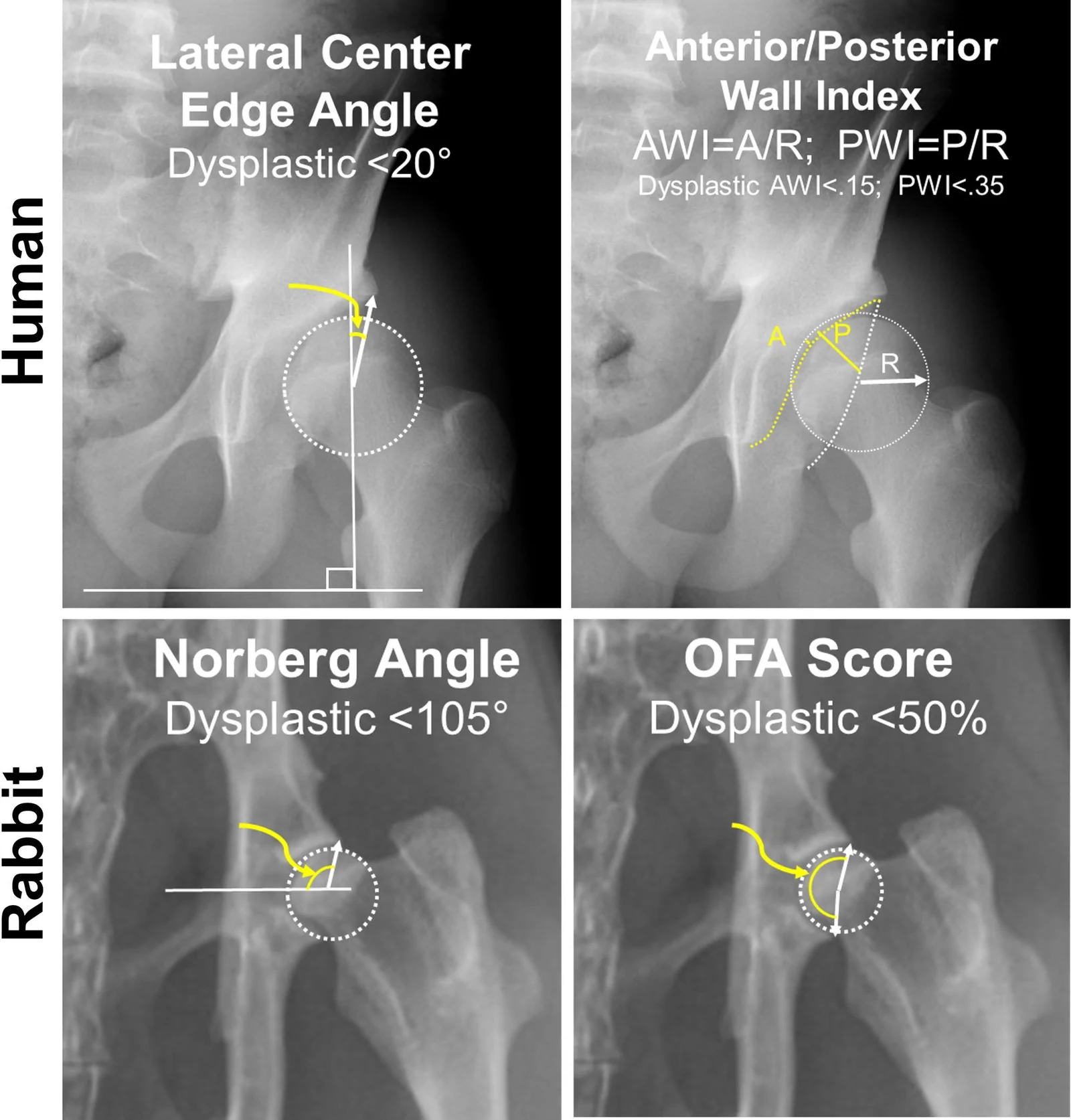
Equivalent radiographic measurements of hip dysplasia can be made in humans and rabbits. The lateral center edge angle in the human is measured nearly identically to the Norberg angle in the rabbit and other quadrupeds, except that lateral center edge is relative to vertical, while the Norberg angle is relative to the horizontal connecting hip centers. The anterior wall index and the posterior wall index quantify the acetabular version component of femoral head coverage by the acetabulum in human hips through the use of linear measures of coverage expressed as a fraction of the femoral head radius. The OFA score in quadrupeds similarly considers the arc of the femoral head covered by the acetabulum, with the angular coverage expressed as a percentage of completely covered (360° around the femoral head)

Fortunately, preclinical animal models of hip dysplasia offer the opportunity to directly assess the natural history of hip dysplasia and the impact that surgical interventions have on joint degeneration. Hip dysplasia in quadrupeds (preclinical modes) is usually quantified with radiographic measurements that parallel those used in diagnosis of human hip dysplasia. The most commonly used clinical radiographic marker for human hip dysplasia after skeletal maturity is the lateral center edge angle of Wiberg (LCEA) [5]. In quadrupeds, LCEA is replaced with the Norberg angle (Fig. 1), which is similarly an angular measurement of acetabular coverage over the femoral head. The key difference is that LCEA measurement in humans is relative to vertical, while the Norberg angle is relative to horizonal and is essentially LCEA plus 90° [6]. The LCEA as a primary assessment of hip dysplasia in humans is often supported by several other radiographic measurements, including the anterior center-edge angle, the Tönnis angle, anterior and posterior wall indices, and extrusion index [7]. Analogous method to quantify hip dysplasia severity in quadrupeds exist, including the Orthopedic Foundation for Animals (OFA) score that quantifies percent of femoral head covered by the acetabular wall on an anterior-posterior equivalent pelvis radiograph (Fig. 1). This is similar to the anterior and posterior wall indices used in the human hip, except the OFA is expressed as an angular percent, whereas the anterior and posterior wall indices are the percent of the femoral head radius covered by the acetabulum [8]. While there are limitations of simple 2D radiographic measures of hip dysplasia, the similarity of measurement techniques that yield values predictive of the development of hip OA in humans and quadrupedal animals illustrate the applicability of preclinical models for the study of hip dysplasia.

Three animal models are experimentally viable to study hip dysplasia and OA development. First, rodents provide a simple, economical medium in which to study hip dysplasia [9–12]. As little as 5–10 days of swaddling neonatal rats with the knee in extension will stimulate developmental dysplasia of the hip [13]. Yet, while there are features of dysplasia in rodents that parallel dysplastic changes in human anatomy, the translational quality of this model is limited due to the thin cartilage of the rodent hip, the physis remaining open throughout the lifespan, and the small size of the hip joint limiting the ability to perform surgical correction of dysplastic deformity. Dogs present a second model system in which to study hip dysplasia, having similar joint structure and vascularity to human hips as well as established surgical treatment of hip dysplasia [6, 14]. Canine hip dysplasia develops spontaneously at incidences up to 50% in high-risk breeds and progresses to advanced OA within a year [6]. However, dogs are an expensive model, and current breeding protocols make it difficult to obtain reliable degrees of dysplastic deformity in sufficient numbers of similarly sized/bred/aged dogs. This results in major heterogeneity of the dysplasia available for study within any given population of dogs.

The rabbit presents a lower expense preclinical model that is viable for both investigating hip dysplasia development and its surgical treatment. While the deformity must be artificially created (it is rarely spontaneous), and the smaller joint size limits the ability to visualize soft tissues using human-equivalent in vivo imaging techniques such as MRI, the rabbit appears to be a useful preclinical study model. Several previous studies have reported various methods to induce hip dysplasia in rabbits, and a selected number of these studies have gone on to assess the impact of surgical interventions on hip OA. This suggests strong potential for deriving useful, translational information from the rabbit model to guide new/evolving treatment of the human condition; however a summary of protocols and results from those studies would be beneficial to guide future hip dysplasia research using rabbits. Thus, the aims of this narrative review were to 1) document protocols used to induce dysplastic deformity in rabbit hips; 2) report radiographic, macroscopic, and microscopic changes in dysplastic rabbit hips; and 3) describe experimental findings of the effects of reorienting osteotomy on joint health that have been generated using this model.

## Main text

### Overview of Previous Literature

We performed a PubMed search in June, 2025 using “rabbit” AND “hip dysplasia” as our search terms. While this was a limited search approach that risks omitting the full breadth of papers available on the topic, it did return a sufficient number of papers to enable this focused narrative review. Any future systematic review or meta-analysis should include additional databases and more comprehensive search terms. Of the 95 papers returned by the initial basic search, we excluded non-English language papers [15–17], reviews not focused on rabbits, studies focused solely on hip blood flow, papers not about rabbits with hip dysplasia, and case reports of dysplastic deformity identified in pet rabbits. This resulted in 21 studies that reported induction of hip dysplasia for scientific study in rabbits [18–38]. Publication dates range from 1962 to 2025 and included a total of 707 rabbits (range 5-101 rabbits in each study) (Table 1). Only three studies were found that evaluated the effects of surgical correction of hip dysplasia on future joint degeneration [27, 29, 31]. One of those studies referenced previous work by the same author group performing a reorienting acetabular osteotomy in rabbits with normal anatomy [39]. This study provides a reference point for interpretation of the reported effects of surgery and illustrates the relative importance of having the dysplastic deformity included as part of the model.

**Table 1:** Summary of different methods for inducing dysplasia in the rabbit

	Induction Technique	Lead Author	Rabbit Age (*n*)	ImmobilizationDuration	Evaluation Timepoint
**Traumatic**	Manual hip dislocation by flexion/adduction	Langenskiöld1962	1–5 days (*n* = 101)	*N*/A	
**Surgical**	Posterior acetabular rim excision	Negri 1977	~ 6 weeks (*n* = 5)	N/A	12 weeks 17 weeks
	Extra-articular bone wedge excised from dorsal acetabulum	Solni1984	﻿3 weeks or 8 weeks (*n* = 18)	N/A	maturity
	Triradiate cartilage ablation	Gepstein 1984	3 weeks (*n* = 30)	N/A	3 weeks 5 weeks 9 weeks
	8 × 4 mm acetabular defect created with rongeur	Gao 2022	3 months (*n* = 8)	N/A	12 weeks 24 weeks
	Ligamentum capitis femoris transection with/without knee splinted in extension	Tomé 2024	6 weeks (*n* = 14)	3 Days	14 weeks
	Ligamentum capitis femoris transection with/without knee splinted in extension	Costa 2025	6 weeks (*n* = 14)	3 Days	14 weeks
**Knee Immobilization In Extension**	Plaster splint; Group with hormone injections added to knee immobilization	Wilkinson 1963	6–8 weeks (*n* = 19)	6 weeks	6 weeks
	Plastic tube Pin in soft tissue posterior to the knee	Michelsson 1972	1–8 weeks (*n* = 72) (*n* = 13)	1 to 8 weeks	Variable
	Metal & plaster splint	Hiranuma 1992	4–6 weeks (*n* = 20)	4 weeks 4 months	1, 3, 6, or 12 months after intervention
	Cast	Li 2010	4 weeks (*n* = 27)	2 weeks 4 weeks 6 weeks	2, 4, 6 weeks 6 months
	Plaster Cast	Zhang 2015	4–5 weeks (*n* = 21)	5 weeks	5 weeks
	Plaster Cast	Wei 2016	4 weeks (*n* = 32)	2 weeks 4 weeks 6 weeks	2 weeks 4 weeks 6 weeks
	Cylinder Cast	Ding 2016	4 weeks (*n* = 24)	8 weeks 10 weeks	8 weeks 10 weeks
	Cylinder Cast	Wang 2018	4 weeks (*n* = 20)	4 weeks	12 weeks after intervention
	Cylinder Cast	Liu 2020	4 weeks (*n* = 80)	8 weeks	8 weeks
	Cast	Ding 2021	4 weeks (*n* = 30)	4 weeks	4 weeks
	Trans-articular K-wire with Cylinder Cast for 1 week	Greenhill 1995	2–3 weeks (*n* ~ 24)	Up to 8 weeks	1 to 8 weeks
	Trans-articular K-wire	Shimogaki 2005	4 weeks old (*n* = 24)	9–14 days	14 weeks 26 weeks
	Trans-articular K-wire	Moraleda 2013	3 weeks old (*n* = 23)	1 week Euthanasia	7 weeks
	Trans-articular K-wire	Zuccon 2022	5 weeks (*n* = 88)	6 weeks	6 weeks

Evaluation Timepoint is relative to the start of deformity development (either surgery or immobilization). In cases which Immobilization Duration match the time listed for Evaluation Timepoint, the animal was immobilized until euthanasia

### Techniques for inducing hip dysplasia in the rabbit

Multiple papers describe traumatic or excisional methods to create dysplastic deformities in the rabbit hip (Table 1) [18–24]. Among the first is a 1962 study that induced hip dysplasia with a traumatic dislocation achieved using a combination of hip flexion, adduction, and a posteriorly directed force in rabbits one to five days old. While 31/101 hips in this series spontaneously reduced, two of the relocated hips and those that remained persistently dislocated developed acetabular and femoral deformities characteristic of hip dysplasia [18]. Other studies describe directly removing portions of the rim [19, 20, 22], ablating the growth plate [21], or transecting the ligamentum capitis femoris [23, 24]. These techniques result in acetabular deficiencies, joint subluxations, and femoral abnormalities which do occur in mechanically unstable dysplastic hips. However, while these induced bone deficiencies may be useful for specific targeted questions related to hip trauma, they poorly represent joint changes arising from instability or abnormal mechanics, and dysplasia created by gradual skeletal remodeling may provide a better representation of the naturally occurring condition for preclinical studies.

**Fig. 2:**
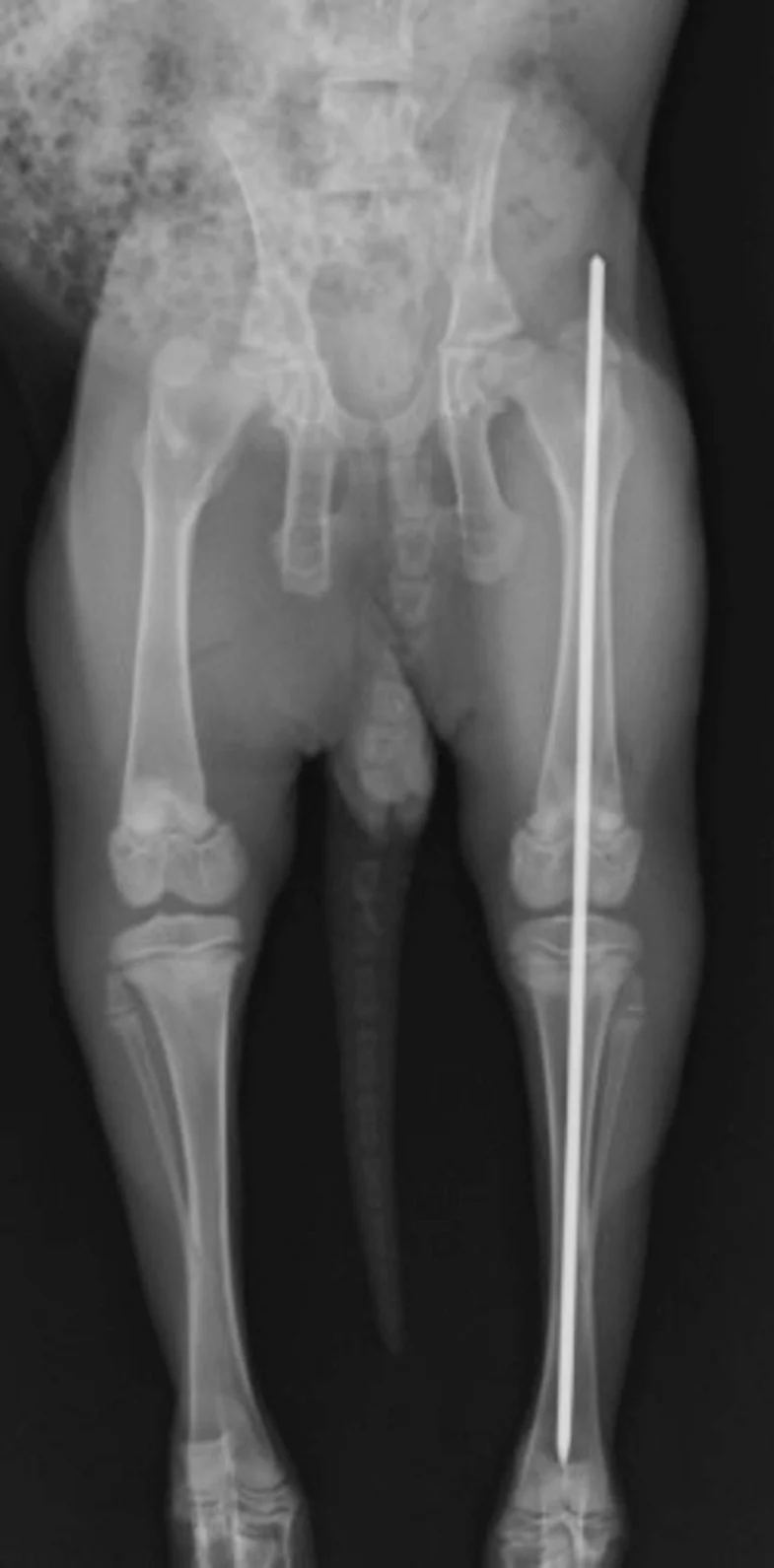
The technique of using a trans-articular K-wire to immobilize the knee in extension. The rabbit shown in this cranio/caudal (anterior/posterior equivalent) is 7–8 weeks of age and a 1.6-mm K-wire was used to span the knee. Michelsson and Langenskiöld first described K-wire immobilization, placing the wire dorsally in the soft tissues of the leg [26]. Greenhill, et al. later modified this technique by passing the K-wire retrograde through the femur, out the greater trochanter, and then distally into the tibia during knee extension [28] as shown here

The most common atraumatic method for inducing hip dysplasia in the rabbit is immobilizing the knee of young, skeletally immature animals in extension. Maintaining the knee in extension sustains elevated posterior/superior (dorsal) stress on the developing acetabulum through the femoral head. Immobilization techniques have included casting [30, 32–37], splinting [25, 27], plastic tubes spanning the knee joint [26], placement of a Kirschner wire (K-wire) either in the posterior leg soft tissues [26] or spanning the knee joint [28, 29, 31, 38], or a combination of methods. No study has reported greater efficacy of any specific immobilization technique. While splinting is a simple procedure requiring relatively fewer resources, splints are bulky and require more ongoing maintenance to manage animals chewing and soiling the splints. Tight splints can cause skin complications along the edges, and a loose splint may fall off or fail to cause the desired hip deformity. In contrast, a knee-spanning, trans-articular K-wire (Fig. 2**)** has the advantage of avoiding skin complications along the edge of the cast or plastic tube, but has the downside of requiring anesthesia for a surgical procedure and fluoroscopy to accurately place the wire across the knee joint.

Rabbit age at the time of knee immobilization ranged from 1 to 8 weeks, with 4 weeks being the most common. The severity of radiographic deformity and reported incidence of subluxation/dislocation is greater with longer periods of immobilization or initiation of immobilization at a younger age [26]. Reported duration of knee immobilization has ranged from 1 to 10 weeks, with longer durations of knee immobilization (3–8 weeks) leading to cases of permanent hip dislocation and shorter durations (< 3 weeks) resulting in more frequent resolution of hip deformity [26, 31]. Generally, immobilization longer than 4 weeks in slightly older rabbits results in persistent features of hip dysplasia (subluxation, increased acetabular index, broken Shenton’s line) without permanent hip dislocation. This severity of dysplasia is most relevant for investigations of hip dysplasia that presents after skeletal maturity, whereas the chronically dislocated models better reflect developmental hip dysplasia in infants and children.

One of the earliest studies describing rabbit models of hip dysplasia used systematic muscle transection at the time of initiating knee immobilization to investigated the mechanism by which extended-knee immobilization drives development of dysplastic deformity [26]. Transecting the hamstrings tendon prevented development of dysplastic hip deformity induced by knee immobilization in all limbs in which it was performed (*n* = 22), while the hips of limbs with the knee immobilized and the quadriceps tendon transected (*n* = 11) developed the dysplastic deformity. Because the hamstring tendon spans both the hip and knee joint posteriorly and is stretched with knee extension, these findings indicate that overtightening of the hamstrings with knee extension is the primary driver of acetabular remodeling and dysplastic deformity development. Muscle-force-driven dysplastic deformity is also seen in humans. One example is in patients with spastic cerebral palsy, where hamstring muscle relaxation via botulinum injection can temporarily prevent progression of hip dysplasia deformity [40]. Population studies of humans that swaddle infants with the hip and knee in extension have higher prevalence of hip dysplasia, likely through a similar muscle tension mechanism [41]. 

### Morphological & radiographic findings

Greenhill, et al. used direct micrometer measurements of dissected dysplastic rabbit hips and found after immobilizing the knee in extension, the acetabulum developed to be 2–3 mm longer in an anterior-inferior to posterior-superior direction than the contralateral hips, and 0.5–1 mm shorter perpendicular to this direction. These changes plateaued 4–5 weeks after knee immobilization [28]. This finding differed somewhat from those of Morelada, et al. in which acetabular diameters were equivalent, but the acetabular depth was significantly reduced in the dysplastic hips [31]. Interestingly, both studies used a trans-articular K-wire to induce dysplasia in 2–3 week old New Zealand White rabbits, but Greenhill, et al. used a longer duration of immobilization (8 weeks vs. 1 week), possibly accounting for more severe acetabular deformity. As neither study reported animal sex, it is not possible to interpret that contribution to the reported acetabular shape differences. Despite the different directions of changes in hip shape, these studies clearly identified changes in acetabular morphology that would result in joint incongruity and instability.

**Fig. 3:**
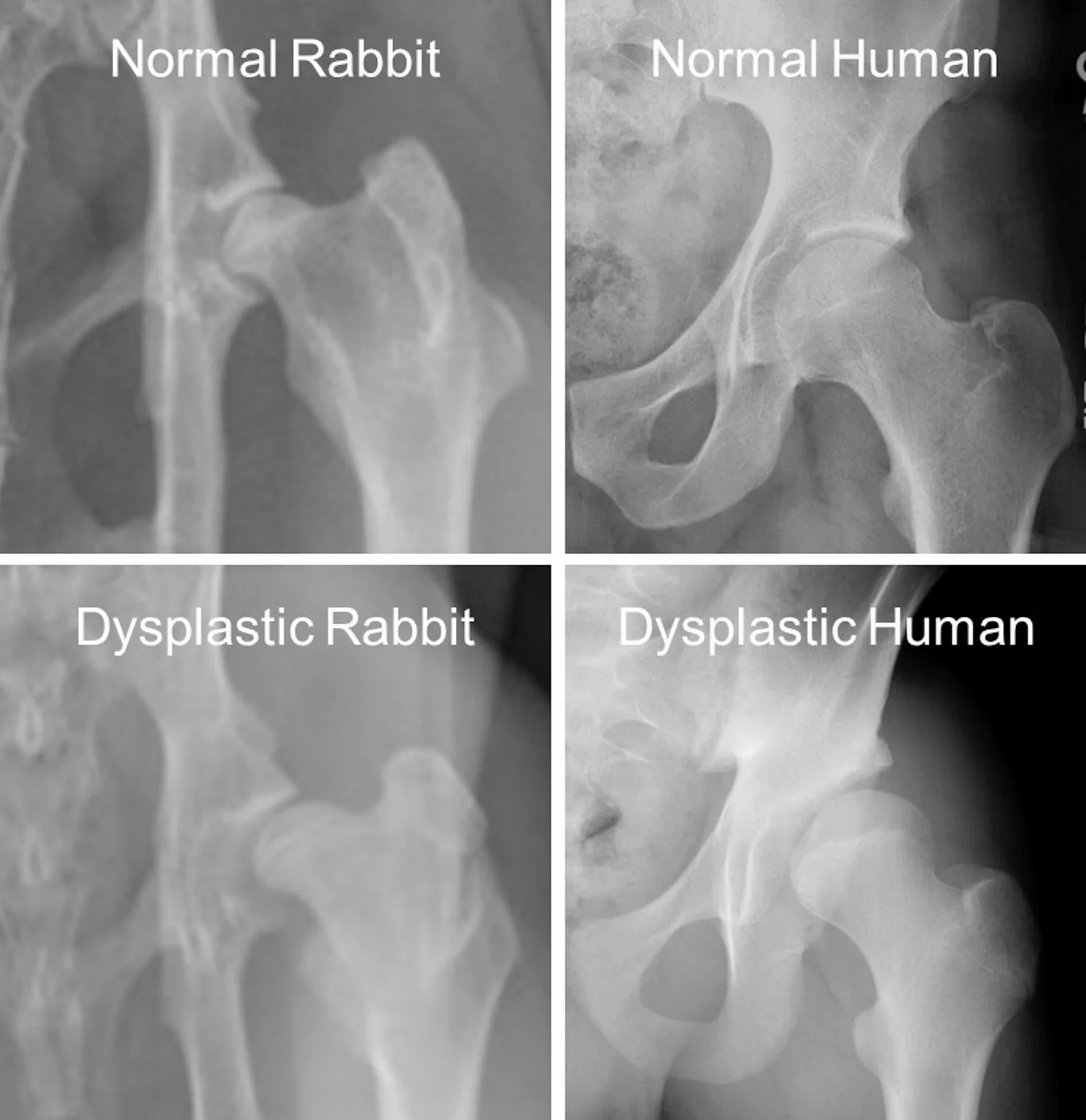
Illustration of common features between dysplastic deformity in human and rabbit hips. While the rabbit joint is smaller than the human joint, similar appearing dysplastic deformity, including upturned acetabular weightbearing surface, lateral subluxation of the femoral head, and reduced joint volume, is present in both species

Most studies rely on radiographs, rather than direct measurements, to assess dysplasia development in the rabbit, finding that dysplastic deformity induced from skeletal remodeling of the acetabulum and proximal femur is qualitatively similar to radiographically apparent hip dysplasia in skeletally mature humans (Fig. 3**)**. The earliest studies qualitatively described radiographically apparent changes in acetabular shape, orientation, and subluxation/dislocation. For example, the study by Michelsson and Langenskiöld reported radiographic findings for both full hip dislocations and dysplastic hips with the femoral head remaining in contact with the acetabulum. Non-dislocated hips developed “sloping of the acetabulum, coxa plana, and coxa vara”, typical radiographic findings of human hip dysplasia diagnosed after skeletal maturity [26]. Other qualitative descriptions of radiographic deformities have included increases in acetabular inclination, an elongated acetabulum, and a discontinuous Shenton’s line [25, 26, 32, 33, 36, 37]. These qualitative descriptions tended to be more common in historical papers, as well as papers in which the primary outcome variables were genetic and cellular markers. Future investigations should include quantitated radiographic measurement values rather than such qualitative assessment of deformity.

The most common radiographic measurements used to quantify hip dysplasia severity in rabbits have been the center edge angle, Sharp’s angle, and the acetabular index (acetabular roof angle/Tönnis angle) (Table 2). Compared to controls, rabbits with dysplasia induced by knee immobilization typically have center edge angle decreases (3° to 10°) [29], Sharp’s angle increases (3° to 5°) [30, 35], and acetabular index increases (ranging 3° to 4° [30, 34, 35], up to 20° to 24° [29, 31]), depending on animal age and duration of immobilization. However, only one of those studies [31], had any measure of interobserver agreement in these small joints. Reports of measurement reliability should be considered an important element for inclusion in future work with this model to allow for better comparison between studies. Other measurements commonly used to assess human hip dysplasia, including the anterior center edge angle, anterior wall index, and posterior wall index are not common due to limitations of radiograph quality and size of the rabbit hip.

**Table 2:** Radiographic findings of hip dysplasia developed in studies using knee immobilization to create dysplastic deformity

Lead Author	Immobilization Technique	Evaluation Timepoint	Acetabular Deformity Developed
Wilkinson 1963	Plaster splint; With/without hormone injections	After 6 weeks of immobilization	• Shallow and deformed acetabular morphology consistent with hip dysplasia • Excessive femoral retroversion and anteversion were observed • Hormone-related changes in laxity affected incidence of dislocation
Michelsson 1972	Plastic tube Pin in soft tissue posterior to the knee	Variable after 1 to 8 weeks of immobilization	• Dislocation when immobilization was performed in younger animals • Dislocation was permanent when immobilization was maintained (3–8 weeks) • Partial or complete resolution of dislocation/dysplasia occurred with older rabbits or shorter durations of immobilization (≤ 3 weeks)
Hiranuma 1992	Metal & plaster splint	3, 6, or 12 months after 4 weeks or 4 months of immobilization	• Subluxation was observed with 3–4 weeks of immobilization • Dislocation occurred in 4 of the hips at 4 weeks
Li 2010	Cast	Immediately or 6 months after 2, 4, or 6 weeks of immobilization	• Acetabular index increased from 27° to 31° after 6 weeks of immobilization • Sharp’s angle increased to ~ 58° compared to contralateral 55° after 6 weeks of immobilization • Acetabular deformity increased with duration of immobilization
Zhang 2015	Plaster Cast	After 5 weeks of immobilization	• Qualitatively described enlarged acetabulum and greater acetabular inclination in 16/21 rabbits • Discontinuous Shenton’s line in 16/21 rabbits
Wei 2016	Plaster Cast	After 2, 4, or 6 weeks of immobilization	• Qualitatively described increased acetabular index and discontinuous Shenton’s line after 2 weeks • Qualitatively described increased acetabular index and discontinuous Shenton’s line with femoral subluxation after 4 weeks • Complete hip dislocation after 6 weeks of immobilization
Ding 2016	Cylinder Cast	After 8 or 10 weeks of immobilization	• Acetabular index increased from 25.4° to 27° after 8 weeks immobilization • Greater incidence of dislocation in 10 week than 8 week immobilization groups
Wang 2018	Cylinder Cast	12 weeks after intervention following 4 weeks of immobilization	• 15/20 (75%) rabbits developed dysplasia and/or subluxation • AI increased from 22° in control to 26° in dysplastic hips • Sharp angles increased from 54° in control to 58° in dysplastic hips • AHI was significantly lower in dysplastic 67% than in control 83% hips
Liu 2020	Cylinder Cast	After 8 weeks of immobilization	• 46/80 rabbits developed dysplastic deformity at 8 weeks • Qualitatively described increased acetabular inclination and discontinuous Shenton’s line
Ding 2021	Cast	After 4 weeks of immobilization	• Non-specific qualitative description of radiographic indicators of dysplasia in 18/30 rabbits
Shimogaki 2005	Trans- articular K-wire	14 or 26 weeks after 9–14 days of immobilization	• LCEA averaged 6° to 7° at pin removal and worsened to 3°/-3° at 14/26 weeks after pin removal • Acetabular index increased from 17°-21° before pinning to 34°/41° at 14/26 weeks after pin removal • 2 hips progressed to dislocation after removal of the immobilization
Moraleda 2013	Trans- articular K-wire	7 weeks after 1 week of immobilization; or after 7 weeks of immobilization	• After 1 week, 7/15 hips developed dysplastic deformity and 6/15 had subluxation • Acetabular index increased from 20° in control hips to 29° in hips with 1 week of knee immobilization and to 44° with 7 weeks of immobilization • Acetabular depth decreased from 5.9 mm to 4.7 mm after 1 week and 4.3 mm after 7 weeks of immobilization
Zuccon 2022	Trans- articular K-wire	After 6 weeks of immobilization	• 19 died, 28 had < 50% subluxation, 41 had > 50% subluxation • 5 rabbits with > 50% had rupture of the round ligament

In humans, CT (computed tomography) is commonly used to assess 3D locations of acetabular coverage deficiency and femoral version in patients with hip dysplasia [42]. Although increased femoral anteversion has been reported in rabbits with induced hip dysplasia [18, 25], this deformity has not been quantified on CT scan. However, two studies have used CT of the rabbit hip to quantify acetabular shape, version, and sector angles, as well as overall pelvis rotation [15, 31]. Although differences among the small numbers of animals included in these studies using CT-based measures were not statistically significant, future work with the rabbit model should still consider assessing hip morphology using cross sectional imaging as it allows for accurate 3D measurements of local coverage and version, as well as longitudinal studies.

### Macroscopic intra-articular findings

Hip dysplasia causes pathologic joint mechanics that directly lead to soft tissue injuries and intra-articular degeneration. Labral tears, articular chondral injury, and partial-thickness ligamentum teres tears are typically seen as a result of hip dysplasia [43–45], and these pathologies are often treated arthroscopically at the time of PAO. However, the impact that addressing labral tears and cartilage defects has on future development of OA is currently unknown.

The rabbit hip dysplasia model also develops macroscopic intra-articular pathology, providing a vehicle for investigators to critically assess the impact of surgically correcting these human-like intra-articular changes. Hypertrophy, lengthening, and tearing of the ligamentum teres is reported in the rabbit model in accordance with magnitude of subluxation/dislocation [25, 31, 34, 38]. Capsular thickening or thinning, dependent on the time immobilized and amount of subluxation, are both reported [34]. Grossly, the acetabular articular cartilage can appear dull, yellow, or abraded [34], and the acetabular rim cartilage also often everts, driving remodeling of the acetabular rim and promoting tearing of the acetabular labrum. Other studies have reported evidence of a neolimbus or formation of a false acetabulum in dislocated hips [31]. However, regular reports of labral pathology in the dysplastic rabbit are sparse. Given how common labral pathology is in human dysplastic hips, detailed reporting using a structured grading scale that documents features such as location around the rim or intrasubstance versus chondrolabral separation-type tears [46, 47] should be included in future studies utilizing the rabbit model.

### Microscopic intra-articular findings

Multiple studies report histopathologic findings in rabbit hips with induced hip dysplasia (Table 3). Most studies of skeletally immature animals present qualitative descriptions of increased cartilage thickness along the acetabular rim, reduced chondrocyte density, loss of chondrocyte organization, and synovial proliferation [28, 29, 32–34]. A recent study by Ding, et al. quantified a 75% increase in acetabular thickness in dysplastic hips compared to contralateral [36]. In contrast, in more skeletally mature rabbits, dysplastic deformity has been qualitatively reported to cause cartilage thinning, surface roughening, fibrocartilage metaplasia, and elevated Mankin scores indicative of osteoarthritis [28–30]. A limited number of electron microscopy studies have reported enlarged collagen fibers and structurally abnormal chondrocytes in dysplastic hips [30, 33, 34], and immunohistochemistry has indicated increased collagen type II expression in cartilage of dysplastic hips [32]. Although these reported microscopic findings generally tend to qualitatively agree across studies, very few groups have quantitated these structural tissue-level features characteristic of dysplastic cartilage in the rabbit hip.

**Table 3:** Reported histologic findings in studies using knee immobilization to create dysplastic deformity of the hip

Lead Author	Immobilization Technique	Evaluation Timepoint	Histologic Observations
Greenhill 1995	Trans- articular K-wire with Cast for 1 week	At weekly intervals from 1 to 8 weeks post immobilization	• **Hematoxylin & Eosin**,** Masson’s Trichrome**: o Qualitative description of cartilage along the posterosuperior acetabular margin in the elongating plane of the acetabulum everting so that the growth front faced away from the center of the acetabulum. o Qualitative description of progressing disorganization of hyaline cartilage in the shortening plane of the dysplastic acetabula and metaplasia to fibrocartilage on the anterior acetabular wall
Shimogaki 2005	Trans- articular K-wire	At 14 or 26 weeks after 9–14 days of immobilization	• **Hematoxylin & Eosin**,** Safranin-O & Fast green**: o Mankin scores of the acetabular cartilage ranged from 5.5–6.5 out of a maximum of 14. o Mankin scores of femoral cartilage ranged from 6–7 out of a maximum 14 (end-stage osteoarthritis) o Qualitative description of articular surface roughening and synovial proliferation
Li 2010	Cast	Immediately after or 6 months after 2, 4, or 6 weeks of immobilization	• **Hematoxylin & Eosin**: o In immature rabbits, qualitative description of acetabular rim cartilage from the dysplastic hip being significantly thicker than that of the normal contralateral. o In mature rabbits, cartilage from dysplastic hips was thinner and more fibrotic than normal contralateral. • **Electron Microscopy**: o The collagen fibrils were enlarged in dysplastic hips compared to normal contralateral. o Chondrocytes in the proliferative cartilage of immature rabbits were more necrotic. o Chondrocytes in mature rabbits with longer casting had more lysosomes.
Moraleda 2013	Trans- articular K-wire	7 weeks after 1 week mobilization; or after 7 weeks of immobilization	• **Masson’s Trichrome & Alcian Blue**: o Qualitative description of cellular disorganization and vascular response to knee immobilization in the triradiate cartilage of dysplastic hips
Zhang 2015	Plaster Cast	After 5 weeks of immobilization	• **Hematoxylin & Eosin**,** Toluidine Blue**: o Qualitative findings that dysplastic cartilage demonstrated surface fibrosis, chondrocyte clusters/clones, and loss of columnar chondrocyte organization • **Immunohistochemical Staining**: o Qualitative description of increased, but uneven type II collagen in the ECM of dysplastic cartilage o Increased expression of type II collagen by chondrocytes in dysplastic cartilage
Wei 2016	Plaster Cast	After 2, 4, or 6 weeks of immobilization	• **Hematoxylin & Eosin**,** TUNEL analysis**: o Qualitative findings of fewer chondrocytes and greater cartilage thickness after 6 weeks (H&E) o Significantly greater number of apoptotic chondrocytes hip cartilage from the 4-week (*p* < 0.05) and 6-week (*p* < 0.01) immobilization groups compared to the control group (TUNEL) • **Electron Microscopy**: o Qualitative finding of progressive chondrocyte shrinkage, nuclear fragmentation, chromatin condensation, and apoptosis with increased duration of immobilization.
Ding 2016	Cylinder Cast	After 8 or 10 weeks of immobilization	• **Hematoxylin & Eosin**,** TUNEL analysis**: o Qualitative description of reduced chondrocyte density, enlarged morphology, and abnormal organization was found in acetabular cartilage of hips with dysplastic deformity (H&E) o Significantly greater number of apoptotic chondrocytes in dysplastic hip cartilage (*p* < 0.001) compared to control group (TUNEL) • **Immunohistochemical Staining**: o Caspase-3 expression was significantly higher (*p* < 0.02), and Bcl-2 expression was significantly lower (*p* < 0.027) in chondrocytes from dysplastic acetabula • **Electron Microscopy**: o Qualitative description of progressive chondrocyte shrinkage, nuclear and chromatin condensation, loss of organelles, and increased presence of vacuoles indicative or chondrocyte apoptosis from dysplasia
Wang 2018	Cylinder Cast	12 weeks after intervention following 4 weeks of immobilization	• **Hematoxylin & Eosin**: o Qualitative description of thinner cartilage, roughened articular surface, and fewer, more disorganized chondrocytes in cartilage from dysplastic acetabula (H&E)
Liu 2020	Cylinder Cast	After 8 weeks of immobilization	• **Hematoxylin & Eosin**,** Safranin-O**: o Qualitative description and representative images of acetabular rim cartilage from the dysplastic hip being significantly thicker than that of the normal contralateral (Saf-O) • **Immunohistochemical Staining**: o Statistically significant downregulation of collagen type X (*p* < 0.05) and Runx2 (*p* < 0.01) in dysplastic acetabular cartilage samples compared to controls, indicating delayed endochondral ossification. o Statistically significant upregulation of GDF11 (*p* < 0.01) and SMAD3 (*p* < 0.05) in acetabular cartilage from dysplastic hips compared to control
Ding 2021	Cast	After 4 weeks of immobilization	• **Hematoxylin & Eosin**: o Acetabular cartilage was significantly thicker (~ 75%; *p* < 0.05) in dysplastic hips than in control hips • **Fluorescent** in situ **hybridization**: o Expression of miR-1-3p was significantly downregulated in the acetabular cartilage from dysplastic hips • **Immunohistochemical Staining**: o Statistically significant increase in Sox9 (*p* < 0.001), decrease in Runx2 (*p* < 0.05), and decrease in collagen type X (*p* < 0.05) in dysplastic hips than in control hips indicating delayed endochondral ossification.
Zuccon 2022	Trans- articular K-wire	After 6 weeks of immobilization	• **Hematoxylin & Eosin**,** Picrosirius Red**: o Fibroblast cell density in the ligamentum teres was significantly higher (*p* < 0.001) in dysplastic hips (average of 6.83%; SD: 3.47%) compared to control (average of 3.87%; SD: 2.13%). o The ligament had significantly more dense collagen (*p* < 0.001) in dysplastic hips than in controls

In addition to altering structure, pathologic loading and unloading of cartilage results in altered chondrocyte metabolic activity, inflammation, and eventual joint degeneration [48, 49]. Combinations of TUNEL analyses and electron microscopy have indicated that 6 weeks of knee immobilization to induce dysplastic deformity significantly increases chondrocyte apoptosis in the acetabular cartilage [33, 34], with the apoptotic state of these chondrocytes supported by quantitated immunohistochemistry [34] and Western blot analyses [33] indicating decreased ratio of Bcl-2/Bax expression and elevated expression of caspases-3 and − 8 (hallmarks of apoptosis). Apoptotic loss of viable chondrocytes reduces production of proteoglycan and extracellular matrix elements needed to maintain cartilage integrity and load-bearing capacity. Other recent studies have quantified increases in gene expression in hips with induced dysplastic deformity and tracked the downstream effectors of those genes. These studies have found evidence of impaired endochondral ossification in the dysplastic hip, which is likely driven by statistically significant upregulations of the SMAD3 and GDF11 pathways, with associated downregulation of both Runx2 and collagen type X, and increases in Sox9 expression [36, 37]. While these specific findings are derived from a rabbit model with the same dysplasia induction technique, and the generalizability of this joint response with other models of induction remains unknown, such data illustrate the ability to directly measure molecular contributors to the formation and resolution of joint pathology that are associated with hip dysplasia in the rabbit model.

### Surgical correction of hip dysplasia

Surgical management of hip dysplasia diagnosed after skeletal maturity has advanced significantly, largely as a result of the 1988 publication by Reinhold Ganz describing early clinical results of hip dysplasia treated with PAO [50]. Yet, despite the broad acceptance of PAO into modern clinical practice, little is known about the impact of acetabular reorientation on degeneration at the cartilage tissue level, or how changes in cartilage health influence progression to osteoarthritis in surgically treated hip dysplasia patients.

The radiographic and morphologic implications of changing acetabular orientation with a combination of surgical procedures and growth modification has been investigated by Moraleda, et al. in the rabbit model [31]. In that work, hip dysplasia was induced by knee extension in three-week-old rabbits, and open epiphysiodesis of the ilioischial limb of the triradiate cartilage was performed at the time of K-wire removal (one week). They found significantly improved lateral coverage of the acetabulum, as measured with LCEA, in rabbits that underwent ilioischial epiphysiodesis. Additionally, the morphology of the acetabulum was improved in the epiphysiodesis group, with histological analyses indicating cellular organization more characteristic of normal joints and more ossification progress in the triradiate cartilage. These findings support the idea that dysplasia can be modulated by surgically altering growth of the acetabulum.

Direct surgical correction of rabbit hip dysplasia has been performed in three previous studies [27, 29, 31], and another study documented histological changes in cartilage after rotational triple acetabular osteotomy (TPO) in non-dysplastic rabbit hips [39]. In the study using rabbits with normal acetabular morphology, a rotational TPO to increase lateral acetabular coverage of the femoral head was performed in sixteen, ten-week-old rabbits and fixed with K-wires. Animals were sacrificed 12 or 24 weeks after the osteotomy. There was no evidence of osteonecrosis or radiographic joint space narrowing at either postoperative timepoint, although the fovea of the acetabulum that was brought over the femoral head demonstrated proliferation of fibrous tissue, and chondrocyte hypercellularity with cloning was observed in the previously unloaded medial edge of the reoriented acetabulum. Mankin scores were substantially higher in the medial acetabulum than other joint locations 12 weeks after osteotomy, although they began to improve by 24 weeks after the previously unloaded cartilage had begun to produce more proteoglycan to support loadbearing and the proliferative chondrocyte cloning response had abated [39]. This responsivity to altered loading in the rabbit hip reflects loading induced changes that are expected in underloaded regions of the human dysplastic hip, and these load-related changes must be kept in mind separately of degenerative changes when interpreting effects of surgical correction in dysplastic hips.

This same research group later published outcomes of performing rotational TPO for 24 rabbits with hip dysplasia induced by 9–14 days of K-wire immobilization of the knee [29]. Two weeks after the K-wire was removed, half of the rabbits underwent TPO. Rabbits were sacrificed 12 and 24 weeks after TPO, or at the equivalent time in rabbits without an osteotomy. Rabbits that did not have TPO had persistent radiographic dysplasia at sacrifice (hip dysplasia did not resolve), while TPO increased lateral coverage and reduced the acetabular roof index. Dysplastic hips that were not treated with a TPO had higher Mankin scores of cartilage degeneration (i.e. greater surface roughness, cellular abnormalities, proteoglycan loss) compared to dysplastic hips treated with osteotomy, although group sizes were small and these results have not yet been repeated. Hips treated with osteotomy were hypercellular and exhibited cloning [29], similar to what was found after osteotomy of a non-dysplastic hip [39]. 

In a different study, Hiranuma, et al. performed a Chiari pelvic osteotomy in rabbits with hip dysplasia [27]. A Chiari pelvic osteotomy medializes the skeletally mature acetabulum (after closure of the triradiate cartilage), thereby repositioning the previously unstable femoral head under the intact ilium. The originating surgeon hypothesized that metaplasia of interposed hip capsule occurs to create a fibrocartilage-lined shelf-type support that constrains the femoral head while maintaining range of motion [51]. To investigate this in the rabbit model, 20 rabbits four-to-six weeks of age had the knee splinted in extension to induce hip dysplasia without dislocation, were aged to skeletal maturity, and underwent a Chiari osteotomy. By 6 months after osteotomy, the newly formed acetabular roof and original acetabulum had smoothly healed on radiographs, and qualitative histological analysis demonstrated that the interposed hip capsule over the medialized acetabulum underwent metaplasia into fibrocartilage with hyaline-like chondrocyte organization and collagen orientation in the weightbearing region [27]. This work is unique because surgical correction was performed after skeletal maturity, whereas other studies focused on treatments prior to skeletal maturity. And while the Bernese PAO has largely replaced the Chiari pelvis osteotomy as the standard-of-care treatment for pre-arthritic hip dysplasia after skeletal maturity, the ability of the rabbit model to parallel human response to hip preservation surgery suggests it is also an appropriate vehicle for preclinical studies of hip osteotomies.

## Conclusions

The rabbit model of hip dysplasia provides a promising platform for evaluating the efficacy of treatment interventions, while also exploring microscopic and macroscopic features of pathology development. Although infants and children with hip dysplasia can present with a complete hip dislocation, adolescent and young adult humans with symptomatic hip dysplasia typically have a femoral head that remains within the shallow, dysplastic acetabulum. Fortunately, use of the rabbit hip dysplasia model provides the opportunity to conduct systematic, controlled studies to fill knowledge gaps that exist across the process of hip dysplasia development and treatment in both skeletally immature and mature animals. Data derived from this model can provide a basis for future studies of joint preservation focused on surgical, biological, or rehabilitative processes, although standardization of induction protocols and more structured or quantitative reporting of outcome measures, including intra and interobserver reliability, are needed to enable comparative analyses across future studies using this model.

## Data Availability

All data summarized in this review are available at the source publications. We have provided full bibliographic details for each reference from which the reviewed data were summarized, including DOIs for those which have digital identifiers.

## Abbreviations

2D: two dimensional
3D: three dimensional
Bax: Bcl–2 associated X protein
Bcl: 2–B–cell lymphoma 2
CT: computed tomography
GDF11: growth differentiation factor 11
K: wire–Kirschner wire
LCEA: lateral center edge angle
OA: osteoarthritis
OFA: Orthopedic Foundation for Animals
PAO: periacetabular osteotomy
Runx2: runt–related transcription factor 2
SMAD3: Mothers against decapentaplegic homolog 3
Sox9: SRY–box transcription factor 9
TUNEL: terminal deoxynucleotidyl transferase dUTP nick end labelling
X: ten
